# Short-Term Nitrogen Enrichment Reshapes Carbon Allocation and Enhances Synergistic Ecosystem Services in Semi-Arid Sandy Grasslands in China

**DOI:** 10.3390/plants14131915

**Published:** 2025-06-22

**Authors:** Litao Lin, Huiyi Yu, Xuekai Sun, Guiyan Ai, Jie Bai

**Affiliations:** 1 Chinese Research Academy of Environmental Sciences, Beijing 100012, China; lin.litao@craes.org.cn (L.L.); yuhuiyi0102@126.com (H.Y.); 2 CAS Key Laboratory of Forest Ecology and Silviculture, Institute of Applied Ecology, Chinese Academy of Sciences, Shenyang 110016, China; awwagy@126.com; 3 College of Environmental Science, Liaoning University, Shenyang 110036, China

**Keywords:** nitrogen enrichment, carbon sequestration, semi-arid grassland, carbon allocation

## Abstract

The capacity to develop resilience to global change, such as nitrogen deposition, is an important topic for the management of key ecological functional zones. In this study, nitrogen enrichment (10 g N m^−2^ yr^−1^, NE) and control plots (0 g N m^−2^ yr^−1^, CL), each with eight replications, were randomly established in the Horqin Sandy Land to investigate how grassland carbon sequestration functions and herdsmen’s livelihoods respond to nitrogen deposition. In addition, three grazing scenarios (non-grazing, light grazing, and moderate grazing) were simulated to determine whether human activities affect the relationships (trade-off vs. synergistic) among forage supply, carbon sequestration, and windbreak and sand-fixing services under nitrogen deposition. The results showed that NE exhibited a significant increase in aboveground carbon storage (99.40 g C m^−2^, 117.34%) and the shoot carbon/root carbon ratio (1.90) when compared to the CL (0.95) (*p* < 0.05). NE significantly decreased soil carbon storage ability, particularly in the 10–30 cm soil layer (*p* < 0.05). The reduction in soil carbon storage was offset by increases in plant carbon storage, resulting in a neutral effect of the NE treatment on the total grassland carbon storage (*p* > 0.05). The synergistic effects of NE on grassland forage supply and windbreak and sand-fixing functions were observed under a light grazing scenario, which balanced ecological safety and livelihood more effectively than the non-grazing and moderate grazing scenarios. These findings indicate that the structure of grassland carbon storage is influenced by nitrogen deposition and that light grazing would enhance ecosystem services and promote sustainable grassland development.

## 1. Introduction

Grasslands cover approximately 37% of the global terrestrial area and comprise one-third of terrestrial carbon sinks, playing a pivotal role in ecosystem services for humanity [1], and are challenged by anthropocentric global changes [2]. As a key driver of global change, nitrogen deposition has raised widespread concern due to its profound ecosystem impacts, particularly for the key ecological function zones, which have emerged as critical central themes in global change research [3]. Many studies have examined how nitrogen enrichment (NE) affects grassland plant diversity, aboveground biomass, and community composition [4,5,6], yet its impact on spatial configuration and multiple ecosystem services remains unclear. The above- and belowground components of grasslands play different functional roles in the ecosystem and are coupled through complex interactions [7,8]. Elucidating how NE affects the vertical allocation of grassland carbon and multiple ecosystem services would therefore contribute significantly to the management and capacity development of grasslands for global change resilience.

Carbon sequestration is a critical ecosystem function and service of grassland ecosystems [9], and it could be affected unequally by NE across vertical layers due to differential allocation among plant shoots, roots, and soil. The aboveground biomass mainly drives photosynthetic fixation and energy flow, whereas belowground biomass (particularly fine roots) mediates nutrient cycling and contributes to soil organic carbon formation through root turnover [8,10]. In a temperate steppe, the root/shoot ratios decreased slightly under NE treatment (0, 1, 2, 3, 5, 10, 15, 20, and 50 g N m^−2^ yr^−1^), with aboveground net primary productivity enhanced by 10–53% and belowground net primary productivity suppressed by 40–47% [7]. Both positive and neutral effects of NE on belowground biomass were observed in some studies [11,12], and this significant variability is highly dependent on interspecific differences in grass functional traits and methodological heterogeneity. Furthermore, the diverse responses of plant root to NE could have a cascading impact on roots and thus affect soil carbon cycling [13]. The nitrogen-mediated soil carbon dynamic would exhibit pronounced variation with depth, governed by the comprehensive influence of root inputs, microbial processing, and mineral protection along soil layers [14,15]. Consequently, studies on carbon allocation among soil organic carbon in different soil layers, root biomass carbon, and aboveground plant biomass carbon are necessary to understand the pathways through which NE affects grassland carbon sequestration and predict the carbon budget under global changes.

Forage supply, windbreak, and sand retention are critical ecosystem services for the agro-pasture ecological zone [1,16]. In general, the forage supply, carbon sequestration, windbreak, and sand-fixing services are closely associated with each other [17]. The amount and quality of the forage supply is positively associated with plant carbon storage and with pastoral livelihoods [18]. Forage harvesting could decrease plant canopy and height [19] and could thus negatively affect the windbreak and sand-fixing service of grasslands [20]. In regions with dense vegetation cover, moderate forage removal may show little impacts on the windbreak and sand-fixing service of grasslands [21,22]. Many existing studies have focused on changes in productivity or carbon sequestration services in response to NE [7,23,24], but the mechanism through which the windbreak and sand-fixing services, as well as their relationship with forage supply and carbon sequestration, respond to NE treatment (i.e., trade-off or synergistic) is not yet understood. Additionally, capacity development for global climate change resilience is also dependent on the herdsmen’s activities (i.e., grazing intensity) [25], which are associated with the income derived from ecosystem services. Property utilization could therefore maximize multiple ecosystem services and harmonize ecological safety and herdsmen’s livelihoods in the grassland ecological zone.

In a sandy grassland ecosystem in Horqin Sandy Land in China, NE trials were conducted with eight replications and corresponding control plots. The objective was to investigate how grassland carbon storage responds to NE treatment under different scenarios. Based on the theory of ecological optimal allocation [8], (1) it was predicted that NE would increase aboveground plant carbon storage more than root carbon storage. Following evidence of high soil respiration in fertilized grasslands [26], (2) it was predicted that NE would reduce carbon density more in the soil surface layer than in the subsurface soil layer and along the soil profile. Additionally, (3) it was predicted that NE would have synergistic rather than trade-off impacts on forage supply, carbon sequestration, and windbreak and sand-fixing services.

## 2. Results

### 2.1. Changes in Plant Carbon Storage with Month, Soil Depth, and NE Treatment

**Figure 1:**
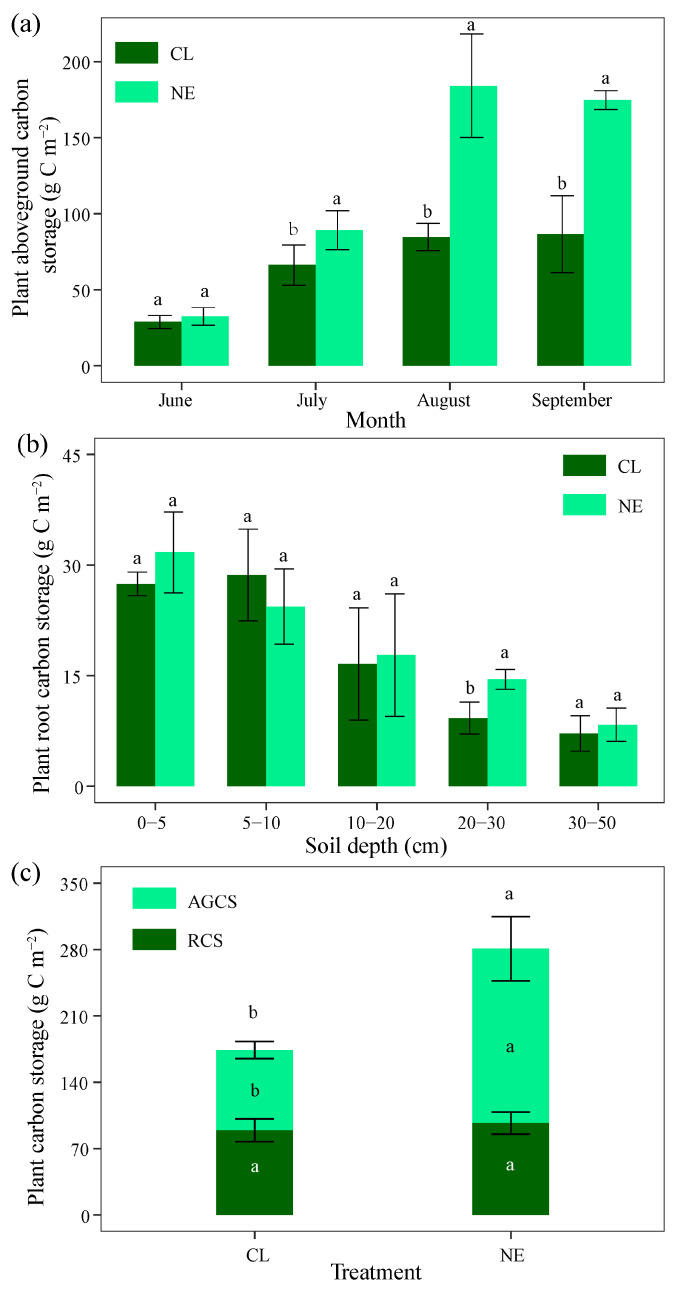
Plant carbon storage in control and nitrogen enrichment plots in a sandy grassland. (**a**) Aboveground plant carbon storage from June to September; (**b**) plant root carbon storage along soil profile in August; (**c**) plant carbon storage in August. CL and NE indicate the control and NE treatments, respectively. AGCS and RCS denote the aboveground plant carbon and root carbon storage in August, respectively. Different lowercase letters represent 0.05 significant differences between CL and NE.

Over a four-month interval, the aboveground plant carbon storage slightly increased during June to August and then remained stable, with the highest storage value (84.71 g C m^−2^) observed during August (Figure 1a, Table S1). The nitrogen enrichment (NE) treatment showed significantly higher aboveground plant carbon storage than the CL from July to September (*p* < 0.05), with increases of 34.69%, 117.34%, and 107.71%, respectively (Figure 1a, Table S2). Across soil profiles, the plant root carbon storage at a depth of 20–30 cm (*p* < 0.05) rather than the other depths (*p* > 0.05) significantly differed between NE and CL plots (Figure 1b, Table S3). Overall, the NE treatment exhibited a greater increase in aboveground carbon storage (99.40 g C m^−2^, 117.34%) than the root carbon storage (7.66 g C m^−2^, 8.58%) and significantly increased the shoot carbon/root carbon ratio (1.90) compared with the CL (0.95) (*p* < 0.05) (Figure 1c, Tables S2 and S3).

### 2.2. Changes in Soil Carbon Storage with Depth

**Figure 2:**
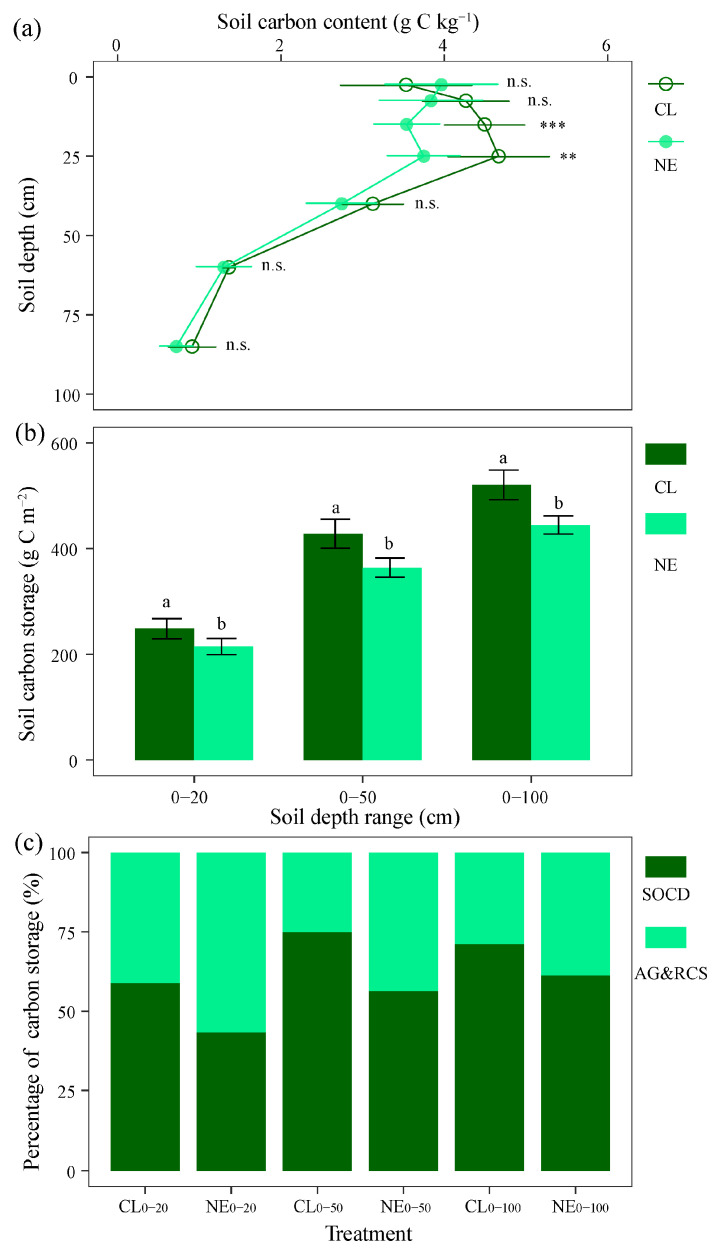
Soil carbon storage in control and nitrogen enrichment plots in a sandy grassland. (**a**) Soil organic carbon content along soil profile; (**b**) soil carbon storage at different depth ranges; (**c**) ratio of soil and plant carbon storage in grasslands. CL and NE denote the control and nitrogen enrichment, respectively. SOCD and AG&RCS denote the soil carbon storage and the sum of the aboveground plant and root carbon storage, respectively. Different lowercase letters represent 0.05 significant differences between CL and NE. **, *p* < 0.01; ***, *p* < 0.001; n.s., *p* > 0.05.

In this study, the soil organic carbon (SOC) content within the 0–100 cm soil profile was examined (Figure 2a, Table S4) and the SOC storage at 0–20, 0–50, and 0–100 cm was 120.16, 299.61, and 391.96 g C m^−2^, respectively (Figure 2b, Table S5). The NE treatment significantly decreased the SOC at depths of 10–20 and 20–30 cm from 4.49 and 4.46 g kg^−1^ to 3.54 and 3.75 g C kg^−1^, respectively (*p* < 0.05) (Figure 2a, Table S5). In addition, the NE treatment significantly decreased the SOC storage at 0–20 (−10.51 g C kg^−1^), 0–50 (−40.66 g C kg^−1^), and 0–100 cm (−52.43 g C kg^−1^) compared with the CL (*p* < 0.05), having a reduction of −8.75%, −13.57%, and −13.38%, respectively (Figure 2b, Table S6).

Considering the SOC storage and plant carbon storage together, grassland carbon storage was 694.11 and 725.39 g C m^−2^ under the CL and NE treatments, respectively. Based on the 0–20 cm depth range, soil and plant accounted for 58.84% and 41.16% of grassland carbon storage, respectively. The NE treatment increased the ratio of plant carbon to grassland carbon storage from 41.16% to 56.68% (Figure 2c). Based on the 0–50 cm and 0–100 cm depth ranges, the NE treatment increased the ratio of plant carbon to grassland carbon storage from 28.88% to 43.56% and from 25.04% to 38.72%, respectively (Figure 2c). The NE treatment thus led to a dominance shift toward the equal importance of plant carbon and SOC storage in grassland carbon storage (Figure 2c).

### 2.3. Changes in Multiple Grassland Services

**Figure 3:**
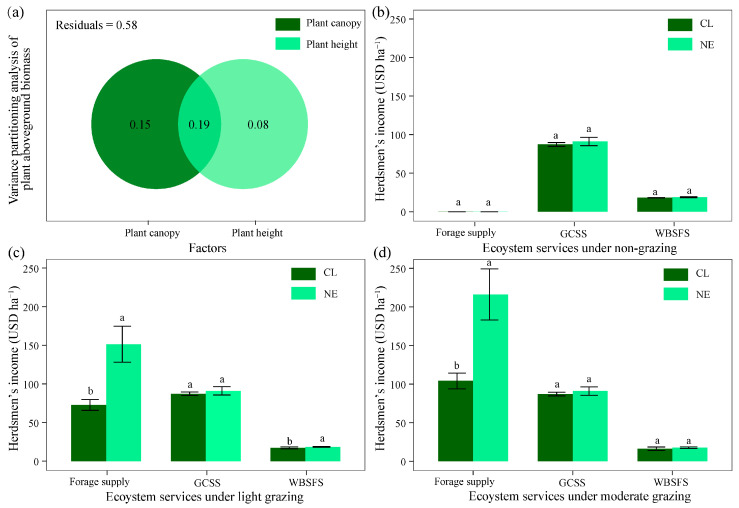
(**a**) Variance partitioning analysis of aboveground plant biomass against plant canopy and height; (**b**) ecosystem service under non-grazing (**c**), light grazing, and (**d**) moderate grazing. CL and NE denote the control and nitrogen enrichment, respectively. GCSS and WBSFS indicate the grassland carbon sequestration service and windbreak and sand-fixing service, respectively. Different lowercase letters represent 0.05 significant differences between CL and NE.

The NE treatment increased the forage supply, carbon sequestration, and windbreak and sand-fixation services from 2.08 t ha^−1^, 6.94 t C ha^−1^, and 0.60 t ha^−1^ to 4.32 t ha^−1^, 7.25 t C ha^−1^, and 0.62 t ha^−1^, respectively, but there were no significant differences (Table 1). To optimize the herdsmen’s profit, this study simulated non-grazing, light grazing, and moderate grazing scenarios to estimate the prices of the forage supply, the carbon pool, and windbreak and sand-fixing services in sandy grassland. The VPA suggested that plant canopy, height, and their joint impact account for 15%, 8%, and 19% of aboveground plant biomass variation, respectively (Figure 3a). The aboveground biomass variation could thus also influence the plant canopy and height after forage removal and the windbreak and sand-fixing service. In the non-grazing and moderate grazing scenarios, the NE treatment did not significantly increase the prices of the carbon sequestration and windbreak and sand-fixing services (*p* < 0.05) (Figure 3b,d). In the light grazing and moderate grazing scenarios, the NE treatment significantly increased the forage supply price from USD 72.86 to 151.33 ha^−1^ (*p* < 0.05) and from USD 104.09 to 216.19 ha^−1^ (*p* < 0.05), respectively. Additionally, the NE treatment significantly increased prices of the windbreak and sand-fixing services in the light grazing scenario compared with the CL (*p* < 0.05), with an increment of 1.17% (Figure 3c). Across the three scenarios, light grazing synergistically increased the prices of the forage supply and the windbreak and sand-fixation services under the NE treatment, and moderate grazing led to the highest total income under the NE treatment (Figure 3).

## 3. Discussion

### 3.1. NE Increased Plant Carbon Storage Above Ground Compared to Below Ground

This study showed that NE significantly increased aboveground plant carbon storage yet showed no significant enhancement in root carbon storage (Figure 1). This result agrees with Liebig’s law of the minimum (i.e., nitrogen limiting) and supported the hypothesis that the aboveground plant carbon storage is more responsive to NE than to root carbon storage. Nitrogen is a critical limiting factor in sandy grasslands due to the poor soil structure and poor soil properties [27], and NE enhances the substance of chlorophyll synthesis and photosynthetic enzyme activity [28], thus increasing aboveground plant carbon storage. Su et al. [14] demonstrated that NE increased leaf nitrogen content by 21%, and Guo et al. [28] reported that Rubisco carboxylation activity in *Stipa grandis* was substantially upregulated under NE treatment (≤50 kg N ha^−1^ yr^−1^, 51–100 N ha^−1^ yr^−1^, and >100 N ha^−1^ yr^−1^), with a 34% increase compared to control conditions. On the other hand, NE may alleviate drought stress by enhancing the concentration of osmotic substances in sandy soils, thereby facilitating plant growth. Lin et al. [29] reported that leaf nitrogen is accumulated to cope with osmotic stress, such as increased salinity and drought. In semi-arid grasslands, fertilized plants could maintain 23% higher water-use efficiency during drought compared to CL, while inducing aquaporin gene regulation and proline accumulation [30]. Consequently, NE significantly enhanced the aboveground plant carbon storage in the sandy grassland by alleviating nitrogen limitation and investing in drought stress response.

The non-significant influence on root carbon storage under the NE treatment could be caused by the optimal allocation of carbon resources. The optimal allocation hypothesis [30] suggest that plants prioritize growth in organs that acquire the most limiting resource (e.g., nitrogen). The acquisition of nitrogen requires substantial energy investment [5], leading to reduced carbon allocation belowground in the NE plots. Su et al. [14] demonstrated that root lignin content (a key structural carbon compound) was decreased by 15% under NE treatment. A 15-year NE experiment in Inner Mongolia steppes also revealed a 137% increase in aboveground biomass but only a 9% increase in root biomass [31]. A meta-analysis of 172 field studies in China showed that NE reduced root-to-shoot ratios by 18–22% in arid grasslands [13]. The disparity between aboveground and root carbon storage stems from the prioritization of carbon allocation to photosynthetic organs under NE conditions (0, 1, 2, 4, 8, 16, 32, and 64 g N m^−2^ yr^−1^) [5]. The NE treatment therefore led to non-significant impacts on root carbon storage and increased the shoot carbon/root carbon ratio in sandy grasslands.

### 3.2. Contrasting Effects of Short-Term NE on Plant and Soil Carbon Storage

Soil organic carbon storage (0–100 cm) significantly decreased by 13.38% under the NE treatment when compared with the CL, with the most pronounced reductions observed in the 10–30 cm layer (−18.40%). The results partially support the hypothesis that surface soil carbon density is more susceptible to NE than subsurface layers. Similarly, in temperate meadow and typical steppes, NE was observed to enhance aboveground biomass, litter production, and shallow root carbon storages (0–10 cm depth) but reduce soil organic carbon storage, lowering the proportion of soil carbon to ecosystem carbon [32]. The decrease in soil carbon storages could be attributed to the decomposition of labile organic carbon and less root exudates under NE treatment. On the one hand, short-term NE (1–3 years) stimulates the microbial activity and decomposition of soil organic matter (SOM). The microbial biomass carbon [33] and soil respiration [26] in sandy soils exhibited positive relationships with soil total nitrogen. The stoichiometric decreases in the soil C:N ratio may trigger the preferential decomposition of lignin-derived compounds [33]. In a *Robinia pseudoacacia* L. plantation, NE (0, 1.5, 3, and 6 g N m^−2^ yr^−1^) reduced labile particulate organic carbon (POC) by 22.5% through enhanced extracellular enzyme activities (e.g., β-glucosidase activity increased by 34%) [34]. The mineral-associated organic carbon (MAOC) remained stable due to its chemical recalcitrance, as observed in Inner Mongolia grasslands where MAOC constituted 68% of total SOC but showed <5% variation under NE [35]. On the other hand, organic carbon at root depth range (e.g., 10–30 cm) was slightly modulated by a reduction in root-derived inputs, such as root exudates [36]. Recent studies have shown that root exudates contribute 20–40% of photosynthetically fixed carbon to rhizosphere microorganisms [23]. More nitrogen available could reduce the amount of root exudates in exchange for soil nutrients [5], thus accelerating the use of organic carbon storage for microbial activities [26]. An agro-ecosystem study demonstrated that NE treatment shifted 23% of surface carbon to deeper layers (40–100 cm) through leaching of dissolved organic carbon [37], and insufficient root exudates in the 10–30 cm layer thus exacerbated carbon depletion.

### 3.3. Synergistic Effects of NE on Grassland Forage Supply and Windbreak Services Under Light Grazing Scenario

The study showed that NE grassland significantly increased forage biomass by 107%, carbon storage by 4.5%, and windbreak service by 3.3% compared to the CL, but there were no differences (Table 1). The result validates the hypothesis that nitrogen enrichment (NE) synergistically enhances carbon sequestration, forage supply, and windbreak and sand-fixing services in sandy grasslands. The synergistically varying pattern could be due to moderate NE enhancing grassland productivity and carbon storage through improved plant growth efficiency [24,38]. Similarly, a global meta-analysis reported that NE increased aboveground biomass by 30–80% and soil carbon by 5–15% [31], consistent with the observed trends. In this study, the increased plant height (27% variance explained) and canopy density (34% variance explained) under the NE treatment improve aerodynamic resistance, reducing wind erosion potential by 1.17% in light grazing scenarios. This structural modification mirrors findings from Inner Mongolia where ecological restoration-induced vegetation thickening reduced dust emission by 49.23% during the period 1990–2015 [39]. Crucially, the absence of nitrogen-induced trade-offs in this study contrasts with temperate grasslands showing nitrogen-driven plant functional trait identity increases at the expense of plant functional diversity loss [4], suggesting that sandy grassland multiple functions and services may possess higher short-term NE tolerance. Nevertheless, prolonged nitrogen loading could alter these dynamics through soil acidification or microbial community shifts [40], necessitating long-term monitoring.

The light grazing (LG) scenario demonstrated an optimal balance in ecosystem service provision and pastoral livelihoods under the NE treatment, generating USD 216.19 ha^−1^ in total service value versus 104.09 ha^−1^ (CL) (Figure 3b–d). The moderate grazing (MG) regime yielded 25% higher total revenue than but similar ecological costs—including suppressed windbreak service (−10.48% in CL and −5.69% in NE)—to the LG regime (−5.58% in CL and −2.68% in NE). Similarly, McSherry and Ritchie [41] found that moderate grazing reduced African savanna carbon storages by 18%, whereas light grazing maintained both productivity and carbon pools that agrees with this study. Li et al. [42] reported that light grazing (30% utilization rate) in Inner Mongolian grasslands maximized live-storage gain and soil retention, agreeing with LG optimization framework in this study. The superiority of the LG scenario aligns with the intermediate disturbance hypothesis, where light herbivory stimulates compensatory plant growth without degrading vegetation structure [43]. On the one hand, partial biomass removal (≤40%) under LG maintains sufficient plant cover (canopy height: *R*^2^ ≤ 0.27, Figure 3a) to protect soil surfaces, whereas MG reduces cover below wind erosion thresholds [16]. The LG scenario facilitates adequate residual biomass for root carbon allocation (7.25 t C ha^−1^ under NE), whereas MG’s intensive defoliation shifts carbon allocation to shoot regrowth at the expense of root reserves [44]. On the other hand, LG concurrently ensures pastoral livelihoods and ecological security, generating USD 151.33 ha^−1^ in forage revenue—38% higher than non-grazing systems (USD 109.92 ha^−1^) (Figure 3c). The LG scenario harmonizes ecological safety (e.g., 3.3% windbreak improvement) with stable income generation, whereas non-grazing systems risk socioeconomic marginalization by eliminating forage revenue without viable alternatives [25].

## 4. Materials and Methods

### 4.1. Site Description and Experiment Design

**Figure 4:**
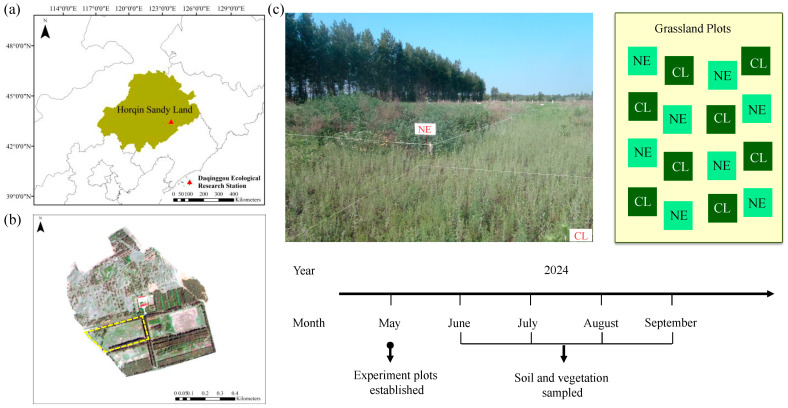
Site location and experimental design of the study. (**a**) Site location of the Daqinggou Ecological Station; (**b**) scan of the grassland ecosystem; (**c**) experimental design of the study. CL and NE denote the control and nitrogen enrichment treatments, respectively.

The research was conducted in the Daqinggou Ecological Research Station (42°58′ N, 122°21′ E, 260 m a.s.l.), in the southeast of the Horqin Sandy Lands, Northeast China (Figure 4a,b). The area is situated in a semi-arid region and characterized by a temperate continental monsoon climate, with a mean annual temperature of about 6 °C, a mean annual precipitation of about 450 mm, and a relative air humidity of about 59% [45]. The frost-free period is about 154 days, and mean annual potential evaporation varies between 1300 and 1800 mm [46]. The soil in the area is characterized as Typic Ustipsamment according to the US Soil Taxonomy designations, developed from eolian parent material [47]. The vegetation was typical grassland, with the dominant vascular species being *Artemisia scoparia*, *Chenopodium acuminatum*, *Setaria viridis*, and *Lespedeza daurica* (Figure 4b,c).

On 3 May 2024, 16 10 m×10 m plots were randomly established in flat and homogeneous sandy grassland, with each plot separated by a 2 m buffer strip (Figure 4c). In each experimental plot, treatments NE (10 g N m^−2^ yr^−1^) and control (0 g N m^−2^ yr^−1^, CL) were performed with eight replications (Figure 4c). For 16 plots, standing dead plants and litter were removed before starting the experiment. In the NE plots, nitrogen was added in the form of urea, which was dissolved in water and sprayed into the plots all at once on 4 May 2024. The CL plots were sprinkled with the same amount of water as the NE plots.

### 4.2. Field Investigation and Sample Collection

From June to September 2024, aboveground biomass was determined by clipping plants within 1 m×1 m quadrats for each plot, with sampling conducted in the middle of each month. Plant samples from each quadrat were oven-dried at 65 °C (72 h) to a constant weight and were weighed in the Daqinggou Ecological Research Station laboratory. In August, following the aboveground biomass collection, soil and root samples were obtained from the same quadrats. Core rings (Φ = 5 cm and V = 100 cm^3^) were used to obtain soil samples at depths of 0–5, 5–10, 10–20, 20–30, 30–50, 50–70, and 70–100 cm for bulk density analyses. Soil cores were collected using a 2.5 cm diameter stainless steel core rings. Soil cores from the same depth were combined as a composite sample and strained through a 2 mm mesh sieve. Soil auger (Φ = 7 cm) was used to obtain soil samples for root extraction at depths 0–5, 5–10, 10–20, 20–30, and 30–50 cm. All roots were thoroughly washed using a 0.3 mm sieve, transferred into small envelope bags, dried to a constant weight at 65 °C (approximately 72 h), and subsequently weighed.

**Figure 5:**
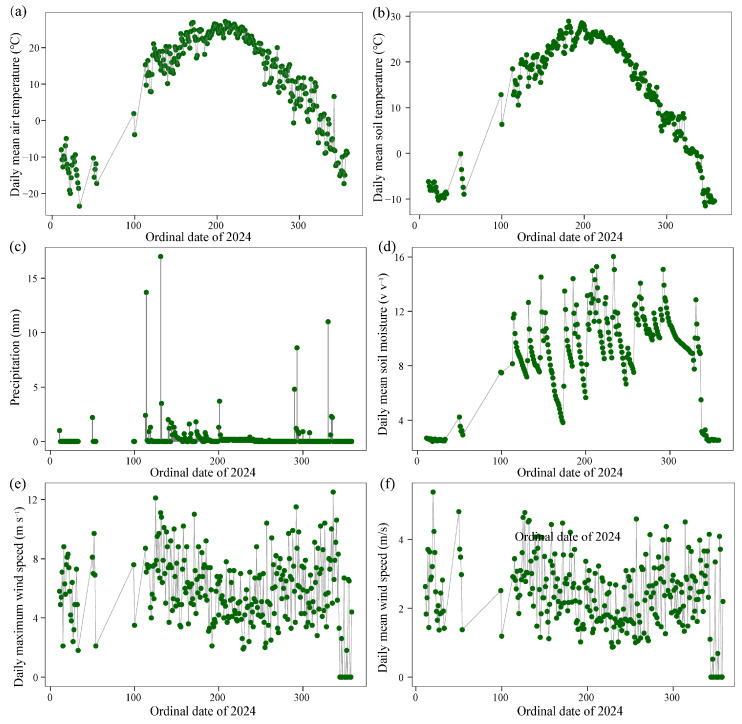
Climate characteristics of the experiment site in 2024. (**a**) Daily mean air temperature at a height 2 m; (**b**) daily mean soil temperature at a depth of 5 cm; (**c**) precipitation; (**d**) daily mean soil moisture at a depth of 0–10 cm; (**e**) daily maximum wind speed at a height of 10 m; (**f**) daily mean wind speed at a height of 10 m.

In 2024, climate parameters (e.g., temperature, wind speed, soil moisture, and precipitation) were recorded hourly by meteorological observation systems (Ausao Ecological Instrument Co., Ltd., Anhui, China) (Figure 5). The air temperature and moisture were measured using a Hygro Thermo Transmitter sensor (Adolf Thies GmbH & Co. KG, Gottingen, Germany). The wind speed at a 10 m height was determined using an Ultrasonic Anemometer 2D sensor (Adolf Thies GmbH & Co. KG, Gottingen, Germany) (Figure 5e,f). Soil moisture was measured by using an ENVILog-100 sensor (Ausao Ecological Instrument Co., Ltd., Anhui, China) (Figure 5d).

### 4.3. Laboratory Analysis of Plant and Soil Samples

Aboveground plant biomass and root biomass were weighed using a balance of one percent (Shangtian Precision Instrument Co., Ltd., Shanghai, China) after being oven-dried at 65 °C. Soil and plant samples were milled to pass through 0.25 mm mesh. The plant organic carbon and soil organic carbon (SOC) were measured. The organic carbon contents of air-dried soil and oven-dried plant material were analyzed via the K_2_Cr_2_O_7_-H_2_SO_4_ oxidation method [48].

### 4.4. Carbon Storage and Ecosystem Service Calculation

Grassland carbon sequestration services (GCSSs) were characterized by the sum of plant and soil carbon storages. To ascertain the carbon storages of the aboveground and root components, the carbon content of each was multiplied by its respective biomass. Soil carbon storage was assessed using the organic carbon density at depths of 0–20, 0–50, and 0–100 cm. The SOC storage at different thickness of soil (0–20, 0–50, and 0–100 cm) was determined by summing SOC densities from each depth. The overall carbon sequestration service was determined by aggregating the carbon storages from both plant and soil components. The overall carbon sequestration service was calculated using the following:

*GCSS* = (*AGCS* + *RCS* + *SCOD*)

*AGCS* = *m*_p_ × *C*_p_

*RCS* = ∑*m*_i_ × *RC*_i_

*SOCD* = ∑*SC*_i_ × *BD*_i_ × *D*_i_ × 10

where *GCSS* is the grassland carbon sequestration service (g C m^−2^); *AGCS*, *RCS*, and *SCOD* are the aboveground plant carbon storage (g C m^−2^), root carbon storage (g C m^−2^), and soil carbon storage (g C m^−2^) values, respectively; *i* is the *i*th depth interval; *m*_p_ and *C*_p_ are the aboveground plant biomass (g m^−2^) and organic carbon content (g C kg^−1^), respectively; *m*_i_ and *RC*_i_ are the root biomass (g m^−2^) and organic carbon content (g C kg^−1^) at the *i*th depth interval, respectively; *SC*_i_, *BD*_i_, and *D*_i_ are soil organic carbon content (g C kg^−1^), bulk density (g cm^−3^), and soil layer thickness (cm) at the *i*th depth interval, respectively.

To estimate the forage supply service, three management scenarios (i.e., non-grazing, light grazing, and moderate grazing) were simulated in the study. According to the FAO, the forage supply could be calculated as the 0%, 35%, and 50% of aboveground biomass, respectively [18]. Additionally, the windbreak and sand-fixing service (WBSFS) of the grassland ecosystem was determined by a modified wind erosion model (RWEQ) [20], which is expressed as follows:

*Q*_sf_ = 0.1699 × (*WF* × *EF* × *SCF* × *K’*)^1.3711^ × (1 − *C*^1.3711^)

*WF* = [∑*WS*_2_ × (*WS*_2_ − *WS*_t_)] × *N*_d_ × *ρ/*(*N* × *g*) × *SW* × *SD*

*ρ* = 348.0 × (1.013 − 0.1183 × *EL* + 0.0048 × *EL*^2^)/*T*

*EF* = (29.09+ 0.31 × *sa* + 0.17 × *si* + 0.33 × *sa/cl −* 2.59 × *OM* − 0.95 × CaCO_3_)/100

*SC* = 1/(1 + 0.0066×*cl*^2^ + 0.21 × *OM*^2^)

*C* = *e*^−0.0483×*SC*^

*K*’ = cosα

where *Q*_sf_ is the amount of windbreak and sand-fixing (t a^−1^); *WF*, *EF, SCF, K’*, and *C* are the climatic erosion factor (kg m^−1^), soil erodibility factor, soil crust factor, surface roughness factor, and vegetation cover factor, respectively; *WS*_2_ represents the wind velocity at a 2 m height (m s^−1^); *WS*_t_ represents the threshold wind speed at a 2 m height (set at 5 m s^−1^); *N* denotes the number of wind speed observations (set at 500 times); *N*_d_ indicates the experimental duration in days (d); *g* is the gravitational acceleration (9.81 m s^−2^); *SW* represents the dimensionless soil moisture factor; *SD* is the snow cover factor; *ρ* stands for air density (kg cm^−3^); *EL* is the altitude (km); *T* is the absolute temperature (in degrees kelvin); *sa*, *si*, and *cl* are the soil sand, silt, and clay content (%), respectively; *OM* is the content of soil organic matter (%); CaCO_3_ is the content of calcium carbonate; *SC* is obtained from measured data of vegetation coverage; and α is set at 0, because the study site is flat.

### 4.5. Statistical Analysis

To determine whether the vertical carbon allocation was unequally affected by NE, two-way ANOVA tests of aboveground plant, root, and soil carbon storages against NE, soil depth or month, and their interactions were analyzed using the aov function in the stats package [49]. To determine the effect of NE on grassland carbon sequestration, forage supply, and windbreak and sand-fixing services, general linear models (GLMs) were conducted by using the lm function in the stats packages [49] and F tests were conducted to measure the significance of the linear regressions. To determine the trade-off or synergistic relationships among ecosystem services, a Spearman correlation analysis was conducted by using the chart-based correlation function in the Performance Analytics packages [50]. A variance partitioning analysis (VPA) was conducted to determine the explanation of canopy density and community height on the aboveground biomass and was calculated by using the varpart function in the vegan package [51]. All the statistical analyses were performed in R 4.4.2 software.

## 5. Conclusions

This study explored the impacts of nitrogen enrichment (NE) on carbon allocation patterns and ecosystem service synergies in semi-arid sandy grasslands. NE significantly elevated aboveground plant carbon storage (117.3%) but exhibited neutral effects on root carbon, increasing shoot carbon/root carbon ratios (1.90 vs. 0.95 in controls), aligning with optimal plant resource allocation theory. While nitrogen enrichment alleviated nutrient limitation to drive photosynthetic carbon gains, it concurrently reduced soil organic carbon storage (−13.4%), particularly in the 10–30 cm layer (−18.4%), attributable to enhanced microbial decomposition and reduced root exudate inputs. Total ecosystem carbon storage remained stable, indicating partial compensation of soil organic carbon loss by vegetation carbon sequestration. Light grazing (LG) synergistically enhanced forage supply (107%) and windbreak and sand-fixing services (3.3%) under nitrogen enrichment, achieving an optimal ecological–economic balance (USD 216.19 ha^−1^ service value), whereas non-grazing and moderate grazing influenced trade-offs. These findings underscore that integrating nitrogen deposition adaptation with LG strategies can stabilize carbon pools while amplifying multifunctional grassland services, providing a sustainable pathway for dryland management under nitrogen depositions.

## Figures and Tables

**Table 1:** Grassland ecosystem services under control and NE treatments. GCSS and WBSFS denote the grassland carbon sequestration function and windbreak and sand-fixing service, respectively. Different lowercase letters indicate 0.05 significant differences between CL and NE. The WBSFS was calculated from June to September.

Ecosystem Service	Price (USD t^−1^)	CL	NE
Forage supply (t ha^−1^)	100.00	2.08 ± 0.20 b	4.32 ± 0.66 a
GCSS (t C ha^−1^)	12.57	6.94 ± 0.19 a	7.25 ± 0.43 a
WBSFS (t ha^−1^)	10.00	0.60 ± 0.01 a	0.62 ± 0.02 a

## Data Availability

The data are contained within this article.

## Supplementary Materials

The following supporting information can be downloaded at: https://www.mdpi.com/article/10.3390/plants14131915/s1, Table S1: Two-way ANOVA of plant aboveground carbon storage against NE treatment and sampling date; Table S2: Plant aboveground carbon storage in the CL and NE plots from June to September; Table S3: Plant belowground carbon storage in the CL and NE plots along soil profiles; Table S4: Two-way ANOVA of soil carbon content against NE treatment and sampling depth; Table S5: Soil organic carbon content in the CL and NE plots; Table S6. Soil organic carbon storage in the CL and NE plots.
