# Supplementary material for: Short-Term Nitrogen Enrichment Reshapes Carbon Allocation and Enhances Synergistic Ecosystem Services in Semi-Arid Sandy Grasslands in China
Source: Plants (Basel). 2025 Jun 22;14(13):1915. doi: 10.3390/plants14131915 (PMC12251850; doi:10.3390/plants14131915)
Supplement: Supplementary file 1 [file plants-14-01915-s001.zip › plants-3688508-supplementary.pdf]

Table S1. Two-way ANOVA of plant aboveground carbon storage against NE treatment and sampling date.

Date	*df*	*SS*	*F value*	*P value*
Treatment	1	45822	159.50	0.000
Date	3	115765	134.32	0.000
Treatment*Date	3	26871	31.18	0.000

Notes: NE, nitrogen enrichment; Date, sampling date; *df*, degrees of freedom; *SS*, sum of squares (SS).

Table S2. Plant aboveground carbon storage in the CL and NE plots from June to September.

Date	AGCS (g m^{-2})		*F* value	*P* value
	CL	NE		
June	28.93±4.30	32.56±5.77	2.03	0.176
July	66.27±13.17	89.26±12.74	12.60	0.003
August	84.71±8.91	184.11±33.96	64.12	0.000
September	86.57±25.29	174.62±6.21	91.44	0.000
All	66.62±27.57	120.14±60.04	159.50	0.000

Notes: AGCS denotes the plant aboveground carbon storage; CL and NE denote the control and nitrogen enrichment, respectively.

Table S3. Plant belowground carbon storage in the CL and NE plots along soil profiles.

Depth	RCS (g m^{-2})		*F* value	*P* value
	CL	NE		
0–5 cm	54.91±3.24	63.49±10.95	4.48	0.053
5–10 cm	57.30±12.46	48.75±10.23	2.26	0.155
10–20 cm	33.12±15.21	25.60±16.60	0.097	0.760
20–30 cm	18.50±4.33	29.02±2.71	34.02	0.000
30–50 cm	14.36±4.80	16.65±4.51	0.973	0.341
0–50 cm	178.19±24.04	193.48±23.35	1.67	0.218

Notes: RCS denotes the root carbon storage; CL and NE denote the control and nitrogen enrichment, respectively.

Table S4. Two-way ANOVA of soil carbon content against NE treatment and sampling depth.

Date	*df*	*SS*	*F* value	*P* value
Treatment	1	3.58	159.50	0.000
Depth	6	187.20	134.32	0.000
Treatment*Depth	6	23.49	31.18	0.001

Notes: NE, nitrogen enrichment; Depth, soil depth; *df*, degrees of freedom; *SS*, sum of squares (SS).

Table S5. Soil organic carbon content in the CL and NE plots.

Depth	SOC (g C kg^{-1})		F value	P value
	CL	NE		
0–5cm	3.53±0.80	3.96±0.69	1.32	0.270
5–10 cm	4.26±0.53	3.84±0.63	2.15	0.165
10–20 cm	4.49±0.49	3.54±0.40	18.13	0.001
20–30 cm	4.46±0.62	3.75±0.46	11.35	0.004
30–50 cm	3.12±0.37	2.74±0.43	3.55	0.081
50-70 cm	1.36±0.07	1.30±0.34	0.24	0.629
70-100 cm	3.19±1.49	2.84±1.31	159.50	0.000

Notes: SOC denotes the soil organic carbon; CL and NE denote the control and nitrogen enrichment, respectively.

Table S6. Soil organic carbon storage in the CL and NE plots.

Depth range	SOCD (g C m^{-2})		F value	P value
	CL	NE		
0–20 cm	120.16±7.53	109.65±7.52	7.80	0.014
0–50 cm	299.61±18.14	258.95±11.62	28.49	0.000
0–100 cm	391.96±20.88	339.53±14.70	33.73	0.000

Notes: SOCD denotes the soil organic carbon storage; CL and NE denote the control and nitrogen enrichment, respectively.
